# The circulating level of interleukins 6 and 18 in ischemic and idiopathic dilated cardiomyopathy

**DOI:** 10.15171/jcvtr.2019.23

**Published:** 2019-06-30

**Authors:** Mahdiyar Iravani Saadi, Mohammad Ali Babaee Beigi, Maryam Ghavipishe, Maryam Tahamtan, Bita Geramizadeh, Abdolhossein Zare, Ramin Yaghoobi

**Affiliations:** ^1^Hematology Research Center, School of Medicine, Shiraz University of Medical Sciences, Shiraz, Iran; ^2^Transplant Research Center, School of Medicine, Shiraz University of Medical Sciences, Shiraz, Iran; ^3^Cardiovascular Research Center, School of Medicine, Shiraz University of Medical Sciences, Shiraz, Iran; ^4^Department of Pathology, School of Medicine, Shiraz University of Medical Sciences, Shiraz, Iran

**Keywords:** Dilated Cardiomyopathy, Heart Failure, Interleukin 6, Interleukin 18

## Abstract

***Introduction:*** By aging population, the heart failure and its life-threatening complications have become an enormous issue in public health. Regarding the inflammation as a major contributing pathological factor, the determination of most important inflammatory targets for immunomodulation is a problematic puzzle in the treatment of heart failure patients and the inflammatory pathways primarily involved in different underlying conditions contributing to heart failure can be an area which is worthy of focused research. Considering the dilated cardiomyopathy (DCM) as a relatively high-incident disease leading to heart failure, the aim of this study is to determine the difference in the expression level of interleukin (IL)-6 and IL-18 in patients with ischemic and idiopathic DCM.

***Methods:*** 39 non-diabetic patients with ischemic and 37 ones with idiopathic DCM were enrolled in the study. 48 healthy individuals were also considered as control group. For quantitative determination of the mRNA expression level of IL-6 and IL-18 genes, an in-house- SYBR Green real-time PCR was used and Glyceraldehyde 3-phosphate dehydrogenase (GAPDH) was considered as internal control gene. The left ventricular end-diastolic volume (LVEDV) and left ventricular ejection fraction (LVEF) was calculated by 2D echocardiographic assessment. Data were finally analyzed via SPSS statistical software version 19.0 using independent t test and 2-∆∆Ct method and *P*<0.05 were considered statistically significant.

***Results:*** The IL-6 was significantly higher expressed in patients with ischemic and idiopathic DCM than in healthy controls (274.3 and 168.8 times, respectively, both *P * values <0.001). The same higher expression of IL-18 was observed in ischemic DCM (48.5 times) and idiopathic DCM (45.2 times) compared with healthy individuals (both *P * values <0.001).

***Conclusion:*** Both ischemic and idiopathic DCM associates with IL-6 and IL-18 overexpression. However, no significant difference was observed between these two subtypes of DCM in either interleukin expression level. There is certainly need to further studies for evaluating the uniformity of results and also assessing other molecules in determining their roles in pathophysiology and probable utility for management.

## Introduction

The heart failure and its morbidity and mortality are a growing concern in current era considering the increased proportion of patients surviving from acute adverse events. In addition, the increased life expectancy and the population aging, both contribute to growing number of patients with heart failure which is a major problem of public health in both developed and developing countries leading to increased health costs associated with the hospitalization and pharmacological or non-pharmacological treatment of patients. Despite the progress achieved in all backgrounds associated with heart failure including its pathophysiology, diagnosis and treatment, the prognosis for patients affected by heart failure has still remained poor. Therefore, the early or on-time diagnosis and the initiation and the establishment of proper and successful treatment in the early stages of disease are important.^1^

Dilated cardiomyopathy (DCM), a leading cause of heart failure, is a relatively high-incident disease, considered as the final response of myocardium to a diverse group of genetic and environmental insults.^2,3^ The most common cause of DCM is ischemic heart disease and among the non-ischemic etiologies, idiopathic DCM is the most prevalent form. The other identifiable causes include those induced by genetic or syndromic disorders, toxins, chronic alcoholism, infections, nutritional deficiencies, endocrine or autoimmune disorders and rarely pregnancy.^3,4^

It is known that different circulating pro-inflammatory biomarkers are increased in patients with both ischemic and non-ischemic cardiomyopathies and their level correlate with severity of disease and prognosis.^5^

The prognosis of DCM has improved greatly during recent decades by etiological classification, earlier diagnosis, more comprehensive approach to nature of the disease, and the optimal pharmacological and non-pharmacological management of various stages of cardiomyopathy. Nowadays, the immunomudulatory therapy has emerged as a possible treatment strategy in patients with heart failure and so, discovering the most important indicators of immunopathogenesis would be helpful considering the probable effect of subgroups of disease.^6^

The aim of this study is to assess the circulating level of interleukin (IL)-6 and IL-18 in patients with ischemic and idiopathic DCM who suffer from heart failure symptoms in order to determine the effects of two main etiologies of DCM on expression of these two interleukins. This study is among initial reports on evaluating the heterogeneity of inflammatory pathways considering various etiological subgroups.

## Materials and Methods

### Subjects

**Figure 1:**
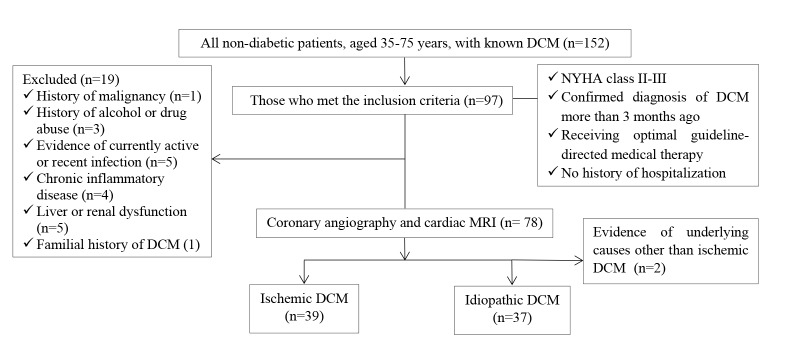
Flow chart of step-wised patient selection.

This case-control study enrolled a total number of 76 non-diabetic patients, aged 35-75 years, with known DCM with NYHA (New York Heart Association) class II-III who were referred to our echocardiography department in Namazee hospital, Shiraz, Iran for follow-up evaluation between February 2016 and January 2017. DCM was diagnosed more than 3 months before the study and meanwhile, all patients received optimal guideline-directed medical therapy and had no history of hospitalization. Of these patients, 39 were affected by ischemic causes designated by angiographic evidence of advanced three-vessel coronary artery disease or thrombosis-induced transmural myocardial infarction and 37 ones were labeled to be suffered from idiopathic DCM after ruling out the possible causes by means of history, physical examination, transthoracic echocardiography, cardiac MRI and coronary angiography. 48 healthy age and sex-adjusted non-diabetic subjects with normal echocardiographic findings were considered as controls. These people were selected among who voluntarily accepted our public announcement about participation in the study. Those with history of malignancy, alcohol or drug abuse, evidence of currently active or recent infection such as viral hepatitis, chronic inflammatory disease including all autoimmune or rheumatologic disease (e.g. Multiple Sclerosis, Inflammatory Bowel disease, Systemic Lupus Erythematosus, etc.) and also those with liver or renal dysfunction were excluded from study considering a comprehensive assessment including meticulous history taking, physical examination and lab investigation (Figure 1).

All patients underwent two-dimensional echocardiographic examination using a vivid E9 system (GE, Norway) and all measurements were performed by one expert echocardiologist according to the latest recommendations of American Society of Echocardiography.^7^ The left ventricular end-diastolic volume (LVEDV) and left ventricular ejection fraction (LVEF) was calculated based on Simpson’s biplane method. For homogenous sampling, the LVEDV index exceeding 100 mL/m^2^ and LVEF between 20-35% were considered as inclusion criteria.

The sample size of at least 28 participants in each group was considered according to such previous studies,^8^ and the type of present study, providing study power of 80% and type I error 5%.

The excluded individuals were substituted by others who met the eligibility inclusion criteria.

### Biochemical assessment

#### Sample preparation

After collecting the Ethylenediaminetetraacetic (EDTA) acid-treated blood samples from each participant, the buffy coat and plasma were isolated by use of Ficoll and then, the samples were preserved in -80°C for further analysis.

#### Ribonucleic acid isolation and cDNA synthesis

Total ribonucleic acid (RNA) was extracted by RNX-Plus solution (CinnaGen, Tehran, Iran). The quantity of extracted RNA was evaluated by NanoDrop 2000 spectrophotometer (Thermo Fisher Scientific, USA) measuring the optical density 260/280, and by running 3 μL of RNA on 1% agarose gel, the quality of RNA was determined by the lack of a smear on the lower part of the gel (presence of a smear indicates RNA degradation) and by the presence of 28S ribosomal RNA (rRNA) twice as intense as of 18S rRNA. After obtaining good-quality total RNA, complementary DNA (cDNA) was synthesized using PrimeScript RT Reagent Kit (Takara Bio, Japan) according to the manufacturer’s guidelines.

#### SYBR green real-time polymerase chain reaction

For quantitative determination of the messenger ribonucleic acid (mRNA) expression level of IL-6 and IL-18 genes, an in-house- SYBR Green Real-Time Polymerase chain reaction (PCR) was used [SYBR^®^ Premix Ex Taq™ II (Tli RNaseH Plus) (Takara, Japan)] applying designed primers specific for each mRNA in iQ5 thermal cycler (BioRad, USA) and Glyceraldehyde 3-phosphate dehydrogenase (GAPDH) was considered as internal control gene. PCR program and primer sequences are summarized in Table 1. Melt curve was analyzed to confirm the specificity of amplification reaction. The results for the target genes were measured as fluorescent signal intensity and normalized to the internal standard gene GAPDH. The changes in the relative expression levels of mRNAs were calculated by 2-∆∆Ct method.^9^

**Table 1:** The biogenetic characteristics of IL-6, IL-18 and GAPDH transcripts

**Gene**	**Primer sequences (5'→3')**	**PCR product length**	**Thermocycling condition**
IL-6:F IL-6:R	ACCCCCAATAAATATAGGACTGGA GCTTCTCTTTCGTTCCCGGT	101 bp	95°C/2 min, 40 cycles of 95°C/30 s, 60°C/20 s and 70°C/30 s
IL-18:F IL-18:R	AGCTTGTGAAAAAGAGAGAGACCT GCTAGTCTTCGTTTTGAACAGTGA	75 bp	95°C/2 min, 40 cycles of 95°C/30 s, 60°C/20 s and 70°C/30 s
GAPDH:F GAPDH:R	GGACTCATGACCACAGTCCA CCAGTAGAGGCAGGGATGAT	119 bp	95°C/2 min, 40 cycles of 95°C/30 s, 57.5°C/20 s and 70°C/30 s

PCR: Polymerase Chain Reaction, F: Forward primer, R: Reverse primer, GAPDH: Glyceraldehyde 3-phosphate dehydrogenase, bp: base pairs.

### Statistical analyses

The statistical differences in expression level of IL-6 and IL-18, and the fold changes in patients and controls were compared via independent t-test and 2-∆∆Ct method, respectively. Statistical analyses were performed with IBM SPSS Statistics for Windows version 19.0 (IBM Corp., Armonk, NY) and p-values less than 0.05 were considered significant.

## Results

The mean age of participants was 52.56 ± 3.7. 44.73% of the patients with DCM and 45.8% of controls were men. Table 2 displays the baseline characteristics of patients and healthy individuals. The functional status and echocardiographic findings of patients with DCM are also summarized in Table 3.

**Table 2:** Baseline characteristics of patients and controls

**Characteristics**	**Ischemic DCM** **(n=39)**	**Idiopathic DCM** **(n=37)**	***P***	**DCM** **(n=76)**	**Control** **(n=48)**	***P***
Demographic data						
Age (y)	54.46 ± 7.2	50.8 ± 12.8	0.126	52.8 ± 10.3	52.4 ± 5.6	0.868
BMI (kg/m^2^)	25.8 ± 2.66	26.1± 3.7	0.684	26 ± 3.41	26.44 ± 5.2	0.931
Gender (Male/n, %)	18 (46.15%)	16 (43.24%)	0.980	34 (44.73%)	22 (45.8%)	0.947
Risk factors					
Smoking (n, %)	8 (20.51%)	7 (18.9%)	0.909	15 (19.7%)	10 (20.8%)	0.935
LDL-C > 130 mg/dL (n, %)	6 (15.38%)	6 (16.21%)	0.829	12 (15.79%)	8 (16.66%)	0.913
TG > 150 mg/dL (n, %)	5 (12.82%)	4 (10.81%)	0.786	9 (11.84%)	5 (10.41%)	0.807
HDL-C < 40 in men or <50 in women (n, %)	3 (7.69%)	2 (5.4%)	>0.999	5 (6.58%)	3 (6.25%)	>0.999
Hypertension	8 (20.51%)	8 (21.62%)	0.828	16 (21.05%)	8 (16.67%)	0.547

Data was expressed as mean ± SD or numbers. The first *P* demonstrates the *P* value of data comparison between two DCM groups and the second *P* denotes the p-value of compared parameters between total DCM patients and control group (t-test was used for comparison of data between ischemic and idiopathic DCM groups and also between total DCM and control groups). DCM: dilated cardiomyopathy, BMI: body mass index, LDL-C: low-density lipoprotein cholesterol, TG: triglyceride, HDL-C: high-density lipoprotein cholesterol.

**Table 3:** The comparison of NYHA class, LVEF and LVEDVI between patients with idiopathic and ischemic dilated cardiomyopathy

**Variable**	**Idiopathic DCM, n=39**	**Ischemic DCM, n=37**	***P*** ** value**
NYHA class III (n, %)	17 (43.58%)	15 (40.54%)	0.971
LVEF (%)	25.7 ± 6.41	28.6 ± 7.85	0.081
LVEDVI (mL/m^2^)	156.87 ± 32.4	148.54 ± 41.57	0.331

Data was expressed as mean ± SD or numbers. DCM: dilated cardiomyopathy, NYHA: New York Heart Association, LVEF: left ventricular ejection fraction, LVEDVI: left ventricular end-diastolic volume index.

**Figure 2:**
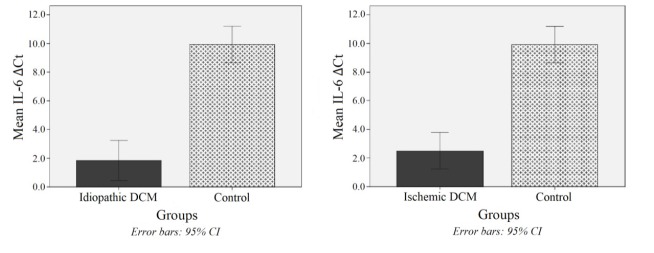
The boxplot of mean IL-6 ∆Ct in patients with idiopathic (Left) and ischemic (Right) DCM both compared with control group (both p-values < 0.001).

**Figure 3:**
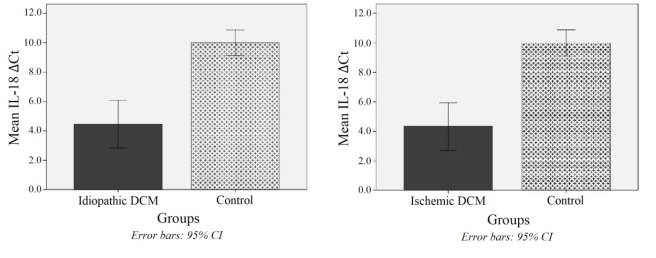
The boxplot of mean IL-18 ∆Ct in patients with idiopathic (Left) and ischemic (Right) DCM both compared with control group (both *P* values < 0.001).

The mRNA expression of IL-6 and IL-18 genes was presented as cycle threshold (Ct) and ∆Ct values. The IL-6 was significantly higher expressed in patients with idiopathic and ischemic DCM than in healthy controls (274.3 and 168.8 times, respectively, both *P* values <0.001) (Figure 2). The same higher expression of IL-18 was observed in idiopathic DCM (45.2 times) and ischemic DCM (48.5 times) compared with healthy individuals (both P-values <0.001) (Figure 3). However, no significant difference was observed between these two subtypes of DCM in either interleukin expression level (both *P* values > 0.999).

## Discussion

As revealed in previous clinical studies, the levels of IL-6 and IL-18 are increased in patients affected by heart failure. Both interleukins activate intracellular signaling pathway and induces various effects on remodeling, hypertrophy and apoptosis.^10^ Similarly, the elevated value of IL-6 and IL-18 was noted in two main subgroups of patients with symptomatic DCM in present study.

In acute decompensation of heart failure, subsequent greater activation of inflammation pathway would be inevitable and so, all enrolled patients here, had no clinical evidence of decompensation in physical examination. This point is valuable because most of the mechanisms of inflammation have been already established under acute setting whether early after tissue injury or during decompensated state and the role of cytokines cascade in the chronic disease and compensated condition has not been fully discovered yet.^11^

A minor trend to worse symptomatic disease as depicted in number of patients with New York Heart Association (NYHA) class III as well as slightly advanced hemodynamic consequences of heart failure as indicated in lower LVEF and higher LVEDV index was seen in individuals with idiopathic DCM; however, this trend was statistically insignificant. More powerfully showed by Hudzik et al,^12^ IL-6 level was inversely correlated with LVEF and predicted the progression of disease. In contrast, a recent study indicated that IL-6 value was not significantly higher in idiopathic DCM patients with poor clinical outcomes (died or underwent transplantation) in comparison to those with better outcomes who were also less likely to be in NYHA class III and to have lower LVEF.^13^

Among other cytokines and inflammatory markers, there is still no strong and uniform association between the level of biomarker and the disease course in trial performed. It has been discovered that some subsets of T cells play important regulatory roles in inflammatory processes involved in pathophysiology of chronic heart failure. Retinoic acid receptor-related orphan receptor-γt (Ror-γt) and also Forkhead box P3 (Foxp3) have been recognized as the “master regulators” of Th17 cells and Treg cells, respectively. On the contrary, anti-inflammatory cytokines such as IL-10 may neutralize inflammation in chronic heart failure. However, as revealed by a recent study, there was no significant difference in FOXP3, RORγt, IL-10 protein expression and supernatant PBMCs IL-10 in patients with chronic heart failure in four functional classes as compared to controls. The level of Foxp3 was significantly lower in chronic heart failure patients with ischemic etiology versus non-ischemic one in the mentioned study.^14^

The informative and practical aspects as well as technical considerations of a group of novel inflammatory biomarkers were assessed in a review concerning their clinical use as prognostic markers in patients with heart failure which revealed that none of the biomarkers evaluated including tumor necrosis factor (TNF) α, the TNF family receptors sTNFR1 and osteoprotegerin, IL-6 and its receptor gp130, the chemokines MCP-1, IL-8, CXCL16 and CCL21 and the pentraxin PTX-3 did fulfill criteria for gold-standard prognostication.^15^

It has been recently noted that immunomodulation is effective in the treatment of heart failure due to viral infection,^16^ and hence, the concept of therapeutic options regarding subtypes of disease requires a precise search for specific probable targets in each subtype, especially those with high potential for better prognosis. Whether a cytokine is a leading cause of initial damage or involved in disease progression, is also of high interest because counteracting it may partly result in disease prevention in subclinical stage or on another extreme, may increase survival or at least quality of life.

Now in modern medicine, there is still insufficient data on the practical use of anti-inflammatory therapies in heart failure. In contrast, established outcome-improving medical treatments of heart failure, including β-blockers, angiotensin converting enzyme inhibitors (ACEIs), angiotensin receptor blockers (ARBs) and aldosterone antagonists seem to at least have some effects on modulating cytokine expression, suggesting further work on inflammatory molecules to guide heart failure therapy.^17^ Even in experimental medicine, there is evidence for anti-inflammatory effects of some complements. For instance, caffeoylxanthiazonoside is an active constituent which is isolated from the fruit of the *Xanthium strumarium* L plant that was shown to have cardioprotective effects on chronic heart failure via inhibition of inflammatory responses in animal models revealing by reduced cardiac hypertrophy and improved systemic ventricle fractional shortening, LVEF and cardiac output associated by suppressing nuclear factor-κB (NF-κB) signaling pathway, decreased level of TNF-α, IL-6 and IL-1β in heart tissues, as well as decreased serum LDH and CK levels.^18^

### Study limitation

In this study, only two interleukins were studied and we confronted a relatively small sample volume due to considering all inclusion and exclusion criteria in just one-year study period. There is need to further multicenter trials of heart failure patients considering different stages of disease to evaluate the effect of disease acuteness and decompensated state on expression of various inflammatory markers. There is also need to consider the large effect of coexisting contributing factors such as acute and chronic comorbidities. The future studies may contribute to certain conclusion about biomarkers of interest and also help investigators find existent cause and effect relationships.

## Conclusion

The IL-6 and IL-18 were higher expressed in both ischemic and idiopathic DCM patients than healthy individuals. However, the circulating level of both interleukins between two groups was not substantially different from each other.

There is certainly need to further studies for evaluating the uniformity of results and also assessing other molecules in determining their roles in pathophysiology and probable utility for management.

## Ethical approval

The study was approved by the Ethics Committee of Shiraz University of Medical Sciences and all patients signed a predefined written inform consent before entering the trial.

## Competing interests

All authors declare no competing financial interests exist.
